# FYCO1 Peptide Analogs: Design and Characterization of Autophagy Inhibitors as Co-Adjuvants in Taxane Chemotherapy of Prostate Cancer

**DOI:** 10.3390/ijms26115365

**Published:** 2025-06-03

**Authors:** Enrico Mario Alessandro Fassi, Roberta Manuela Moretti, Marina Montagnani Marelli, Mariangela Garofalo, Alessandro Gori, Cristiano Pesce, Marco Albani, Erica Ginevra Milano, Jacopo Sgrignani, Andrea Cavalli, Giovanni Grazioso

**Affiliations:** 1 Department of Pharmaceutical Sciences, Università degli Studi di Milano, Via L. Mangiagalli 25, 20133 Milano, Italy; marco.albani@unimi.it (M.A.); ericaginevra.milano@unimi.it (E.G.M.); giovanni.grazioso@unimi.it (G.G.); 2 Department of Pharmacological and Biomolecular Sciences, Università degli Studi di Milano, Via G. Balzaretti 9, 20133 Milano, Italy; roberta.moretti@unimi.it (R.M.M.); marina.marellimontagnani@unimi.it (M.M.M.); 3 Department of Pharmaceutical and Pharmacological Sciences, Università di Padova, Via F. Marzolo 5, 35131 Padova, Italy; mariangela.garofalo@unimi.it (M.G.); cristiano.pesce@phd.unipd.it (C.P.); 4 National Research Council of Italy, Istituto di Scienze e Tecnologie Chimiche (SCITEC-CNR), Via M. Bianco 9, 20131 Milano, Italy; alessandro.gori@icrm.cnr.it; 5 Institute for Research in Biomedicine (IRB), Via Chiesa 5, 6500 Bellinzona, Switzerland; jacopo.sgrignani@irb.usi.ch (J.S.); andrea.cavalli@irb.usi.ch (A.C.)

**Keywords:** peptide, LC3B binders, autophagy, cancer, Atg8, LIR motif, FYCO1

## Abstract

Autophagy plays a central role in cellular degradation and recycling pathways involving the formation of autophagosomes from cellular components. The Atg8 protein family, particularly LC3, is essential to this process, and dysregulation has been implicated in many diseases (including cancer). Furthermore, therapeutic strategies targeting Atg8 proteins like LC3 can be advanced by exploiting the expanding knowledge of the “LC3 interacting region” (LIR) domain to develop inhibitory ligands. Here, we report a computational approach to design novel peptides that inhibit LC3B. The LIR domain of a known LC3B binder (the FYCO1 peptide) was used as a starting point to design new peptides with unnatural amino acids and conformational restraints. Accomplishing molecular dynamics simulations and binding free energy calculations on the complex of peptide–LC3B, new promising FYCO1 analogs were selected. These peptides were synthesized and investigated by biophysical and biological experiments. Their ability to affect cellular viability was determined in different cancer cell lines (prostate cancer, breast cancer, lung cancer, and melanoma). In addition, the ability to inhibit autophagy and enhance the apoptotic activity of Docetaxel was evaluated in PC-3 prostate cancer cells. In conclusion, this research presents a rational approach to designing and developing LC3B inhibitors based on the FYCO1-LIR domain. The designed peptides hold promise as potential therapeutic agents for cancer and as tools for further elucidating the role of LC3B in autophagy.

## 1. Introduction

Within living organisms, autophagy operates as a meticulously orchestrated mechanism, targeting specific proteins and aged or impaired organelles through the use of double-membrane vesicles termed autophagosomes. Upon the fusion of autophagosomes with lysosomes, the enclosed contents undergo degradation facilitated by the acidic milieu and lytic enzymes contained within the lysosomes [1,2]. The capacity for recycling within the autophagy machinery is found not only in eukaryotic cells but also in bacteria, enabling the preservation of physiological conditions [2,3]. The autophagy machinery comprises over 50 proteins named Atgs, with those belonging to the Atg8 family primarily responsible for autophagosome formation and cellular trafficking. In mammals, Atg8 proteins (mAtg8) consist of two subfamilies: GABARAP (GABA-A receptor-associated protein) and MAP1LC3 (microtubule-associated protein 1 light chain 3), also known as LC3. The GABARAP subfamily encompasses GABARAP, GARAPL1, and GABARAPL2, whereas the LC3 subfamily includes LC3A (comprising two splicing variants, LC3Aα and LC3Aβ), LC3B, LC3B2, and LC3C [4]. Proteins belonging to the same subfamily demonstrate notable sequence resemblances and fulfill analogous roles within the cellular environment. The GABARAP subfamily is pivotal in autophagosome closure and the enlistment of autophagic components, whereas LC3 proteins primarily engage in cargo recruitment during the process [2]. The exact impact of compounds that disrupt Atg8 proteins on autophagy remains incompletely understood. However, utilizing peptides or peptidomimetics to inhibit the Atg3–Atg8 interaction in *Plasmodium falciparum* holds promise as a strategy to combat malaria [5,6]. Conversely, it has been confirmed that dysregulations of the complex autophagy machinery are associated with diseases like neurodegenerative disorders [7], cardiomyopathies [8], infectious diseases [6,9], type II diabetes mellitus [10,11], hepatic steatosis [12], and cancer [13,14,15]. These conditions arise from the dysregulation of autophagy induced by various stimuli originating from internal or external environmental factors.

The function of autophagy in cancer appears to be highly complex and may have opposite roles in different cancer cells, stages, and conditions. In normal cells, autophagy removes altered molecules or dysfunctional organelles, maintaining cellular health. Furthermore, autophagy plays a protective role by maintaining genome stability and reducing cellular alterations involved in cellular transformation. For this reason, a reduction in autophagy activity is observed in the early stages of tumorigenesis. Conversely, in the advanced and metastatic stages of cancer, autophagy increases, allowing tumor cells to survive and adapt to foreign sites. In addition, autophagy is activated in cancer cells exposed to various stresses such as anticancer treatments, leading to chemoresistance [16,17].

Specifically, LC3B is significantly upregulated in prostate cancer (PC) tissues, especially in metastatic castration-resistant PC (mCRPC), as compared to benign prostate tissues [18,19]. In PC tissue, high-level LC3B expression is associated with key clinicopathological indicators of aggressive disease, including high Gleason scores and advanced tumor grades, highlighting its involvement in tumor progression and aggressive growth [19]. In addition, a lack of immunoreactivity for LC3B is an independent predictor of PC specific mortality, indicating that autophagy is complex and context-dependent in PC evolution [18]. Moreover, because taxane chemotherapy induces cellular stress that often triggers autophagy as a protective mechanism in tumor cells, targeting LC3B-mediated autophagy represents a promising adjuvant strategy to sensitize PC cells to taxanes, potentially enhancing treatment efficacy and reducing recurrence rates [20].

In this complex context, numerous studies have been conducted in PC evaluating the effects of autophagy activators, such as Rapamycin, Everolimus, and Temsirolimus, or inhibitors, such as Chloroquine (CQ) and hydroxychloroquine (HCQ), alone or in association with conventional therapies [21,22] to better understand the impact of autophagy on cancer progression.

These agents serve as invaluable tools for delving into the complex mechanisms of autophagy on a molecular scale. Furthermore, there exists potential for their further advancement into promising drug candidates, with the aim of dealing with pathological conditions such as cancer and other related medical ailments [23]. In this field, in our previous paper [24], we utilized computational methods to design peptides called WC8 and WC10, which demonstrated both a high calculated and measured affinity for GABARAP. Intriguingly, when prostate cancer cells (PC-3) were treated with WC8 and WC10 at concentrations ranging from 1 to 10 µM, the attained results highlighted the significant therapeutic potential of this approach. Notably, the peptides exhibited greater activity compared to Paclitaxel, a widely used anticancer drug [24]. Nevertheless, LC3B remains the most extensively studied Atg8 protein in humans since it is clearly associated with cancer.

Interestingly, the proteins involved in the autophagy process and capable of interacting with LC3 feature a distinct amino acid sequence referred to as the “LC3 interacting region” (LIR). This small protein sequence consists of four conserved residues that can be succinctly represented as a sequence of “X_0_–X_1_–X_2_–X_3_”, where X_0_ represents an aromatic residue (Trp/Phe/Tyr), X_1_ and X_2_ can denote any amino acids (often acidic or hydrophobic residues), and X_3_ signifies a large hydrophobic residue such as Leu, Val, or Ile [25]. Consequently, the LIR domain can be considered a promising starting point to design a ligand capable of interacting with the LC3 subfamily. For this reason, among the proteins bearing the LIR domain, we focused our attention on the “FYVE and coiled-coil protein 1” (FYCO1), a protein involved in the transport of autophagosomes along microtubules in the plus-end direction [26,27]. In more depth, FYCO1 is an adaptor that connects LC3B on autophagosomal membranes to Rab7 and phosphatidylinositol-3-phosphate (PI3P), allowing for coordinated movement along the cytoskeleton and facilitating the microtubule plus-end-directed transport of autophagic vesicles. Rab7 binding facilitates vesicle docking and fusion with lysosomes, while its interaction with LC3B through the conserved LIR motif guarantees selective recruitment to autophagosomal membranes. Through PI3P interaction, the FYVE domain of FYCO1 maintains membrane association, and its coiled-coil region facilitates microtubule motor recruitment and dimerization, both of which are necessary for directional transport. FYCO1 behaves as a crucial regulator of autophagosomal maturation and intracellular trafficking due to its dual binding to LC3B and Rab7, which guarantees the effective lysosomal degradation of cargo [27]. Effective cargo degradation is made possible by LC3B binding, which stabilizes FYCO1′s association with autophagosomal membranes and guarantees appropriate vesicle docking and fusion with lysosomes. This interaction’s specificity keeps autophagosomes from mislocalizing, preserving the integrity of intracellular trafficking during autophagy. Moreover, FYCO1 plays a crucial role in LC3-associated phagocytosis by being recruited to Dectin-1 phagosomes and aiding in their maturation, transitioning them from early p40phox-containing phagosomes to late LAMP1-positive phagosomes [28]. In addition, FYCO1 and protrudin collaborate to promote the microtubule-mediated transport of late endosomes through endoplasmic reticulum–endosome contact sites [29]. Cerulli et al. [30] employed the FYCO1-LIR peptide to conduct structure–activity relationship (SAR) studies, systematically investigating how to enhance its preferential binding affinity and selectivity for LC3B over GABARAP. In particular, they systematically deleted some residues and identified the crucial determinants of the binding, e.g., the *N*-terminal region, E1287, critical hydrophobic interactions. Artificial amino acids (e.g., 1-naphthylalanine at position F1280 and tert-butylalanine at L1288) further enhanced binding to hydrophobic pockets. This improved affinity was also due to the introduction of an *N*-terminal arginine. Their effort resulted in a peptide (Comb1) with a 2.4-fold increase in binding affinity vs. the original FYCO1 peptide, as well. Indeed, the researchers also used diversity-oriented stapling to improve both the stability and efficacy of their peptides, culminating in novel inhibitors of LC3B [30].

Here, adopting the affordable computational protocol reported in our previous papers [24,31], we have designed new 12-residue-long FYCO1 peptide analogs capable of inhibiting LC3B. The newly designed FYCO1 analogs are shorter than the ones reported by Cerulli et al. [30] but, similarly, contain unnatural amino acids as well as conformational rigidification. All peptides were simulated in complex with LC3B protein by molecular dynamics (MD) simulations and the peptide binding free energy values were predicted by the Molecular Mechanics–Generalized Born surface area (MM-GBSA) approach, aiming to select the most promising FYCO1 analogs to be subsequently synthesized and tested by biophysical and biological in vitro assays on cancer cell lines.

## 2. Results and Discussion

### 2.1. Computational Design of FYCO1-LIR Analogs

FYCO1, a protein composed of 1478 amino acids, binds the LC3A and LC3B protein, principally by means of the amino acids belonging to the LIR domain, especially by the sequence FDIITDEE (1280–1288 region) [26], as demonstrated in the X-ray structure of the human LC3B in complex with the LIR domain of FYCO1 (DAVFDIITDEEL, PDB accession code 5D94 [26]). Here, we used this domain as the starting point to design new FYCO1 analogs, aiming at computationally designing new peptides endowed with improved affinity for LC3B. These new peptides, could weaken the maturation process of LC3B to LC3-II, finally shaping the autophagosome.

In our approach, we optimized the FYCO1 sequence by the rigidification of the peptide backbone and incorporating unnatural amino acids to limit the protease liability of the new peptides. In particular, the FYCO1-LIR domain (DAVFDIITDEEL) was initially simulated in complex with LC3B by accomplishing energy minimization and MD simulations. When the peptide reached geometrical stability in complex with LC3B, the peptide binding free energy value (ΔG*) was calculated by the MM-GBSA approach, attaining a value of −110.6 kcal/mol (Table 1). This value served as a reference for the subsequent design steps.

### 2.2. Design of FYCO1 Analogs

**Figure 1:**
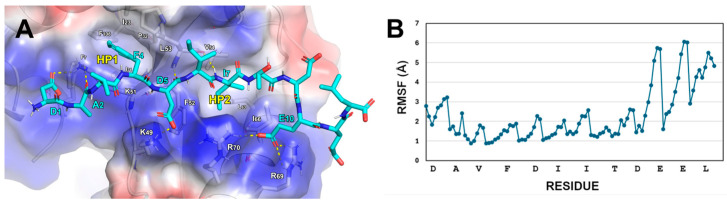
(**A**) Predicted binding mode of FYCO1-LIR (cyan sticks) in complex with LC3B resulting at the end of the MD simulation. The protein surface is colored depending on the atomic partial charges of the protein residues: blue for positive- and red for negative-charge areas, respectively. The H-bonds are represented as yellow dotted lines. (**B**) RMSF plot of FYCO1-LIR no-H heavy atoms (highlighted as dots).

Analysis of the final frame of the LC3B/FYCO1-LIR complex MD trajectory, through visual inspection, highlighted several key interactions (Figure 1A):

- The D1 of FYCO1-LIR establishes contact with K51 on LC3B.
- FYCO1-LIR’s F4 residue engages in cation-π stacking with the side chain of LC3B-K51. Its phenyl ring is also positioned within the hydrophobic pocket 1 (HP1) on LC3B, formed by the residues F7, I23, P32, I34, L53, and F108.
- A hydrogen bond is formed between the NH group of D5 in FYCO1-LIR and the carbonyl group of LC3B-K51. Additionally, the acidic tail of D5 creates a salt bridge with LC3B-K49.
- The side chain of I6 in FYCO1-LIR is solvent-exposed, while the side chain of I7 projects inward, interacting with the hydrophobic pocket 2 (HP2) on LC3B, which is composed of the amino acids I35, F52, V54, L63, I66, and I67.
- The residue E10 of FYCO1-LIR interacts with the side chains of R69 and R70 on LC3B.

The root mean square fluctuation (RMSF) plot of the FYCO1-LIR heavy atoms (Figure 1B) indicated that the *C*-terminal region of the reference peptide does not strongly bind to the LC3B surface but, interestingly, the LC3B-HP2 pocket surrounding FYCO1-LIR I7 appeared to have some extra space, and this led us to consider replacing I7 with a bulkier amino acid like methionine (**AM1** peptide, Table 1), potentially leading to a more tightly packed structure.

To validate this hypothesis, we simulated the **AM1** peptide in complex with LC3B, observing a predicted ΔG* value almost 3 kcal/mol lower than that of the parent peptide, though the RMSF value was comparable to that of FYCO1-LIR (Table 1). Building upon this, we reasoned that the LC3B-HP1 pocket, which binds F4 of FYCO1-LIR, could potentially accommodate a larger hydrophobic side chain. To test this, we simulated two unnatural peptides, **AM2** and **AM3**, incorporating bromo- and iodo-phenylalanine at position 4. The growing applications of bromine and iodine substituents in anticancer treatment lend support to their selection. By inducing mitochondrial apoptosis and activating MAPK pathways, bromine-containing compounds, like bromamine T [32], have shown strong cytotoxic effects against breast and colon cancer cells, exhibiting superior anticancer activity when compared to non-halogenated analogs. Furthermore, because bromine and iodine isotopes can produce deadly Auger electrons that destabilize cancer cells at a very short range, they are being investigated in radiopharmaceuticals for targeted cancer therapy [33]. Notably, only **AM2** exhibited a lower predicted peptide binding free energy value and a significant reduction in the overall peptide conformational fluctuation (see RMSF values, Table 1). Integrating all these findings, we designed and simulated a novel peptide, **AM4**, incorporating both bulkier residues targeting LC3B-HP1 and HP2 pockets. Interestingly, the predicted ΔG* and RMSF values for **AM4** were approximately 13 kcal/mol and 0.36 Å lower, respectively, than those of FYCO1-LIR (Table 1). These results suggested that the concurrent optimization of bulky side chains at positions 4 and 7, to enhance interactions with HP1 and HP2, yielded the most promising peptide among those designed up to that point.

### 2.3. Design of Stapled Peptides

We also sought to enhance peptide structural stability and reduce *C*-terminal RMSF by introducing covalent crosslinks. Disulfide bonds, a common strategy for restricting conformational freedom and stabilizing secondary structure in peptides, often improve drug-like properties and metabolic resistance. This approach aimed to enhance binding affinity, selectivity, cell permeability, and proteolytic degradation resistance [34,35,36]. Given the spatial proximity of D9 and L12 in FYCO1-LIR during MD simulations of the LC3B/**AM4** complex, we substituted these residues with cysteines to create a disulfide bridge, resulting in the **AM5** peptide. Subsequent simulation of **AM5** in complex with LC3B revealed a comparable predicted ΔG* value, but a markedly lower average Cα atom RMSF (Table 1).

Building upon our previous designs, the peptide **AM6** incorporated both the productive I7M mutation from FYCO1-LIR and the conformational rigidification provided by the disulfide bonds in **AM5**. Remarkably, the simulation of **AM6** in complex with LC3B yielded a new low predicted ΔG* value (Table 1). To explore the impact of linker length, we designed **AM7**, a stapled peptide with a -CH_2_-Ph-CH_2_- spacer connecting the sulfur atoms of C9 and C12. MD simulations indicated that this modification improved the predicted ΔG* value by approximately 8 kcal/mol compared to FYCO1-LIR, while concurrently enhancing the structural stability of the peptide (Table 1). Taking into account all these data, we extended our simulations to analogs incorporating iodo- and bromo-phenylalanine at position 4 (**AM8** and **AM9**, respectively), while maintaining the **AM5** scaffold. This approach allowed us to assess the impact of halogen substitution on the peptide’s conformational behavior and the interaction network. The attained results suggested that this modification significantly increased the structural stability of the peptides, as indicated by the reduced RMSF values (≈1 Å, Table 1). However, in both cases, the binding affinity toward LC3B remained largely unchanged compared to the native FYCO1-LIR peptide (Table 1).

**Figure 2:**
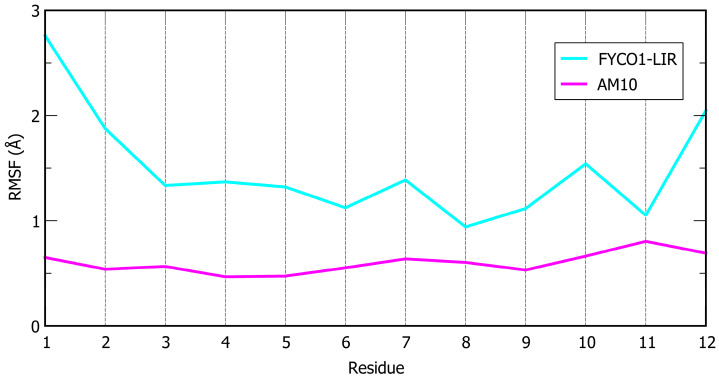
Cα atoms RMSF plot of FYCO1-LIR (cyan line) and **AM10** peptides (magenta line).

In a final design iteration, we combined the most advantageous individual substitutions, namely, the I7M mutation (as in **AM6**) and the incorporation of iodo-phenylalanine at position 4 (as in **AM8**), within the **AM5** scaffold, yielding the **AM10** peptide. This peptide exhibited the lowest predicted ΔG* (−137.1 kcal/mol) and Cα RMSF (0.60 Å) values among all designed peptides, representing the most promising candidate. All these findings indicate that individual modifications are insufficient to substantially enhance peptide performance. However, the combination of all modifications exerts a strong synergistic effect, resulting in a peptide in which all residues are stably anchored on the LC3B protein surface. In fact, the calculated RMSF, with all values significantly below 1 Å (Figure 2), indicates minimal fluctuations. This stability is further corroborated by the RMSD analysis, which reveals that this peptide maintained a remarkably lower average deviation compared to all other designed peptides throughout the simulation (Supplementary Materials, Figure S1).

**Figure 3:**
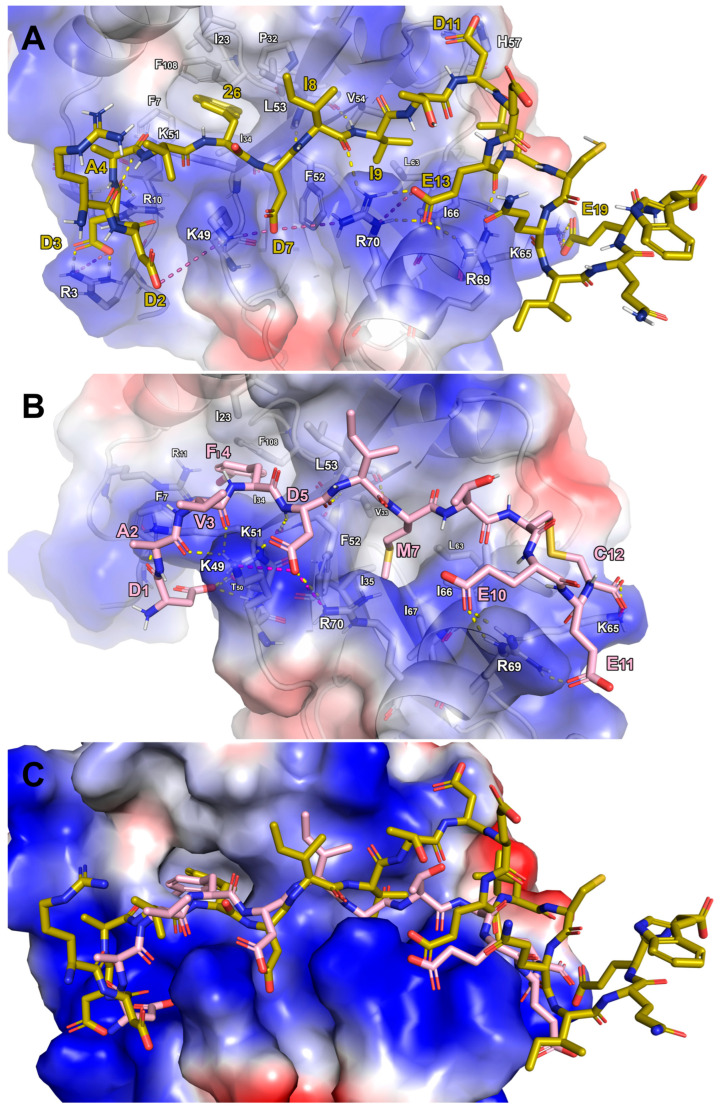
Predicted binding mode of (**A**) Comb1 (sand sticks) and (**B**) **AM10** (pink sticks) in complex with LC3B resulting in the most stable RMSD conformation during the MD simulation. (**C**) Superposition of the Comb1 and **AM10** binding mode on the LC3B protein surface. The protein surface is colored depending on the atomic partial charges of the protein residues: blue for positive- and red for negative-charge areas, respectively. The H-bonds and salt bridges are represented as yellow and purple dotted lines, respectively. The label sizes are proportional to the distance from the viewpoint.

To compare our findings with Cerulli et al.’s 2020 study, we conducted docking and MD simulations on Comb1 (sequence RDDAV2DIITDEEαCQIQEW, in which “2” denotes 2-naphthylalanine and “α” is a tert-butylalanine), one of most potent and LC3B-selective peptides [26]. Structural alignment of the MD-stabilized LC3B-Comb1 complex with the LC3B/**AM10** complex revealed key interaction similarities (Figure 3). Specifically, the *N*-terminal residues of both peptides were located in an LC3B region enriched with positively charged residues, facilitating the formation of electrostatic and H-bond interactions. Of particular note is that the interaction sites involving LC3B-K49 and K51 are common to both peptides. However, only Comb1 engages H-bonds with R3 and R10, whereas **AM10** uniquely forms two H-bonds with T50. The 2-naphthylalanine (residue “2” in the sequence) of Comb1 at position 6, projected into the LC3B-HP1 hydrophobic pocket, was structurally mimicked by the Iodo-F4 of **AM10**. Furthermore, Comb1 exhibited similar interactions to **AM10** at several key sites: D7 and E13 of Comb1 mirrored D5 and E10 of **AM10** in their interactions (H-bonds/salt bridges) with LC3B-K49, -R69, and -R70. Comb1-E19 interacted with LC3B-K65 similarly to the C-terminus of **AM10**-C12. Additionally, I9 of Comb1 occupied the LC3B-HP2 region analogously to **AM10**-M7. However, a key difference was the deeper penetration of **AM10** into the hydrophobic pocket, allowing for additional hydrophobic contacts with LC3B-I35 and -I67.

In conclusion, computational analyses suggested that the shorter decapeptide **AM10** can emulate the interaction patterns of the longer Comb1 eicosapeptide. In addition, **AM10**′s cyclic conformation imposes geometric rigidity, enabling side chains to adopt conformations that maximize binding interactions through spatially matched residue complementarity.

In summary, our computational investigations have identified novel FYCO1 analogs possessing improved predicted binding affinity (lower ΔG* values), enhanced stability on the LC3B surface, and increased metabolic stability through the incorporation of disulfide bonds and unnatural amino acids.

### 2.4. Synthesis of Peptides

Based on the computational outcomes, the **AM2**, **AM6**, **AM7**, and **AM10** peptides were selected for synthesis and biophysical analysis (Table 1), while FYCO1-LIR was chosen as a control. In addition, these peptides incorporate key mutations that provide valuable insights into structure–activity relationship (SAR) studies. They were assembled by conventional solid-phase peptide synthesis (Fmoc-) and HPLC-purified before further processing. Disulfide cyclic analogs were obtained by H_2_O_2_ oxidation in mild conditions, whereas covalent cysteine crosslinking was induced by mixing the linear peptide form with the corresponding di-bromol linker (**AM7** peptide) in aqueous NaHCO_3_/acetonitrile buffer (pH 8.0). Upon full conversion into their macrocyclic forms (<1 h, HPLC monitoring), the resulting compounds were HPLC-purified (see the Materials and Methods for details).

### 2.5. Biophysical Assays

**Figure 4:**
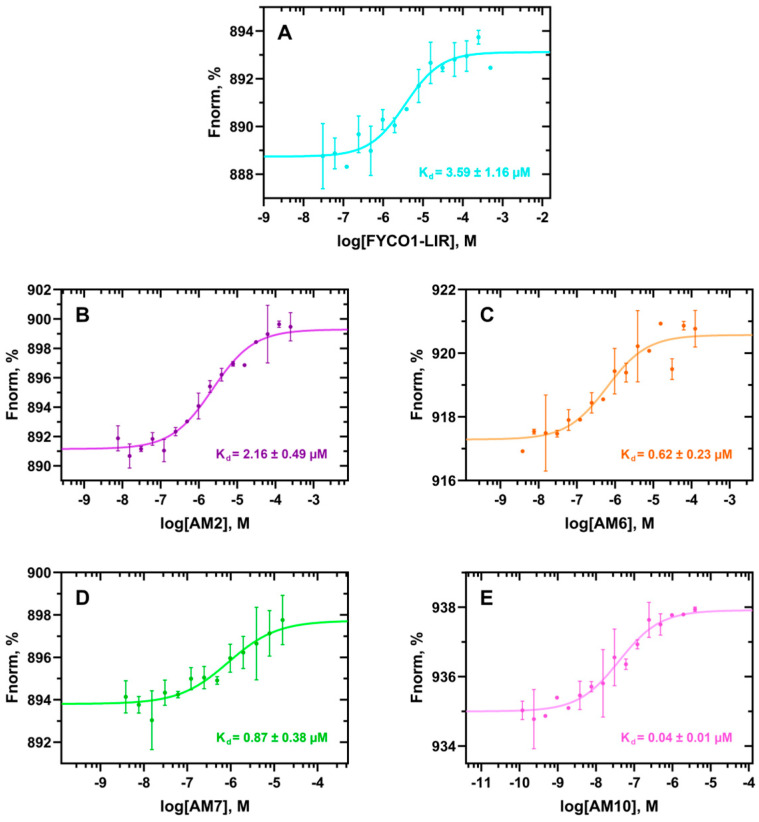
MST curves obtained by incubating human recombinant His-tagged LC3B protein with different concentrations of the control peptide FYCO1-LIR (**A**) and **AM2** (**B**), **AM6** (**C**), **AM7** (**D**), and **AM10** (**E**), using the Monolith NT.115^Pico^ instrument. In the case of **AM7**, the three highest concentration points (125, 62.5, and 31.25 µM) were discarded due to aggregation. (*n* = 2 independent measurements; error bars represent the standard deviation).

To measure the dissociation constants (K_d_) of the peptides on human recombinant His-tagged LC3B protein, MST experiments were carried out on a Monolith NT.115^Pico^ instrument (see the Materials and Methods section for details). To validate the applied biophysical method, the K_d_ value of the FYCO1-LIR peptide (DAVFDIITDEEL) was used as a positive control, yielding a K_d_ value of 3.6 ± 1.2 µM (Figure 4A). This result closely aligns with the values reported by Cerulli et al. [30], who measured the K_d_ of FYCO1 (DDAVFDIITDEELW) using Biolayer Interferometry (BLI) assays, obtaining a value of 3.1 ± 0.6 µM [30]. To note, the slightly lower K_d_ value observed by Cerulli and coworkers could be explained by the presence of additional D and W residues in the amino- and carboxy-terminal groups, respectively, in the FYCO1 sequence tested by them. Next, the binding affinity of the peptides **AM2**, **AM6**, **AM7**, and **AM10** to the human LC3B protein was assessed. Interestingly, in the case of **AM2**, the addition of an iodine atom to the benzyl group of F4, which is projected into the HP1 pocket, nearly halved the K_d_ value (2.2 ± 0.5 µM, Figure 4B) compared to FYCO1-LIR (Figure 4A). In addition, from the MST experiments, it is clearly observable that the iodine group of **AM2** stabilizes the complex; in fact, the points of the K_d_ curve fit much better, and this is reflected in a high signal-to-noise ratio (SNR) value (14.4, Supplementary Materials, Table S1). Interestingly, these results reflected both the predicted computational ΔG* and RMSF values for **AM2** (Table 1).

The binding affinity to the LC3B protein significantly improved for the peptides with backbone rigidification and I7M mutation (i.e., **AM6** and **AM7**), as shown by their K_d_ values of 0.6 ± 0.2 µM and 0.9 ± 0.4 µM, respectively (Figure 4C,D). Interestingly, in this case it is also observable that there is a good correlation between the computationally predicted ΔG* and experimental K_d_ values (Table 1). The MST experiments reveal that a disulfide bond (**AM6**) facilitates binding interactions more effectively than a bulkier linker bridging the sulfur atoms (**AM7**). Furthermore, the -CH_2_-Ph-CH_2_- linker displays aggregation at concentrations of 31.25 µM or higher (Figure 4D), interfering with the MST analysis. The reliability of computational studies is further confirmed by MST analysis of the stapled peptide with the lowest predicted ΔG* and RMSF values (**AM10**, Table 1). Notably, this peptide exhibited the highest binding affinity for human LC3B protein (K_d_ = 0.04 ± 0.01 µM). This finding highlights the crucial role of the iodine group in F4 and demonstrates that its combination with other mutations, such as M7 and C9–C12 (involved in a disulfide bond), leads to a dramatic improvement in K_d_. In fact, the K_d_ value is about 90-fold lower than that of the parent peptide FYCO1-LIR.

### 2.6. Biological Assays on PC-3 Cells

**Figure 5:**
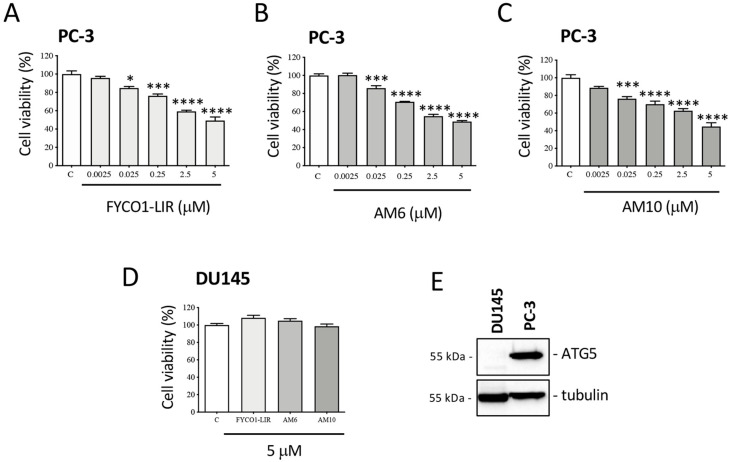
Effect of FYCO1-LIR analogs on CRPC cell viability. (**A**–**C**) PC-3 cells were treated with FYCO1-LIR, **AM6**, and **AM10** for 72 h. Cell viability was evaluated by MTT assay. (**D**) DU145 cells were treated with FYCO1-LIR, **AM6**, and **AM10** for 72 h. Cell viability was evaluated by MTT assay. Data represent the mean values ± SEM of six biological samples (n = 6) and were analyzed by one-way analysis of variance ANOVA followed by Dunnett’s post hoc test (* *p* < 0.05 vs. C; *** *p* < 0.001 vs. C; **** *p* < 0.0001 vs. C). (**E**) Analysis of ATG5 expression in DU145 and PC-3 cell lines. Tubulin expression was evaluated as a protein control.

Based on the biophysical results, the biological activity of the peptides FYCO1-LIR (used as a reference), **AM6**, and **AM10** was evaluated on the viability of two CRPC cell lines which differ in their ability to activate the autophagic process: PC-3 cells, which show measurable endogenous autophagic activity; and DU145 cells which, due to lacking the ATG5 protein, are unable to form autophagosomes and activate the autophagic process [37]. PC-3 cells were treated for 72 h with increasing doses of FYCO1-LIR, **AM6**, and **AM10**, and at the end of the treatment, an MTT assay was conducted. Figure 5A–C shows that all peptides reduce cell viability in a significant dose-dependent manner starting from the 0.025 mM dose up to the 5 mM dose. Treatment of DU145 cells with the same compounds for 72 h at a dose of 5 mM showed no effect on cell viability (Figure 5D). This result highlights that the compounds are specific and selective; in fact, they are ineffective in DU145 cells that are ATG5-deficient, as shown in Figure 5E.

**Figure 6:**
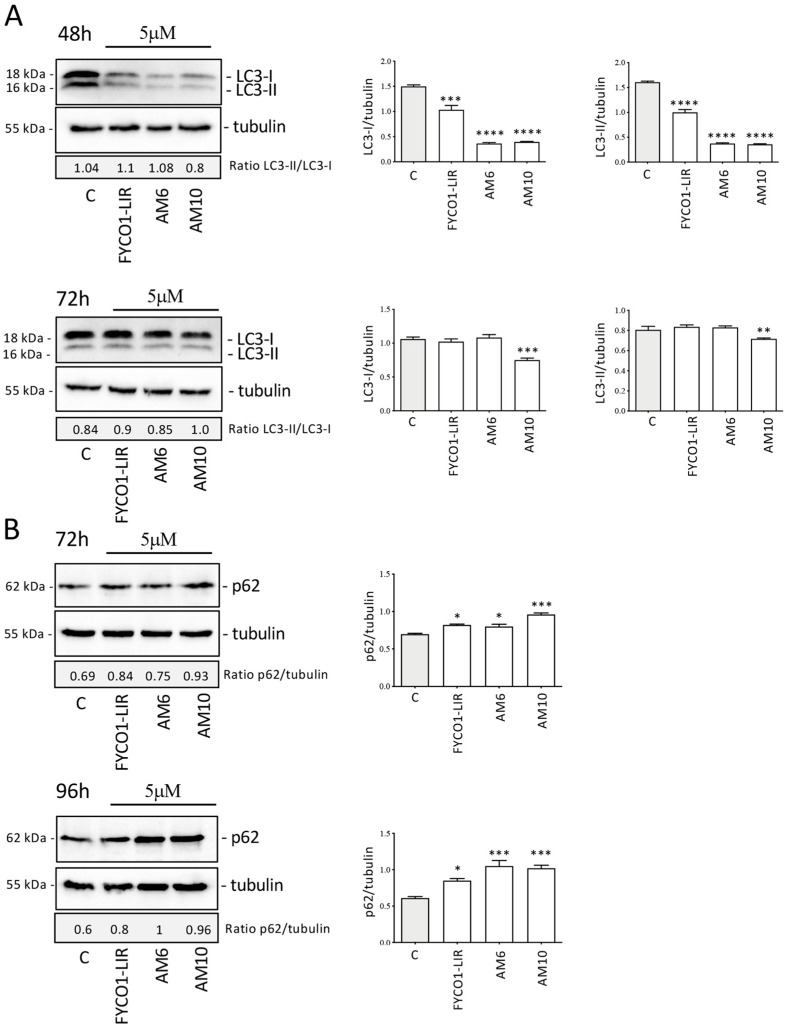
Effect of FYCO1-LIR analogs on autophagy in PC-3 cells. (**A**) Analysis of LC3 expression after treatment with FYCO1-LIR, **AM6**, and **AM10** (48 h and 72 h). (**B**) Analysis of p62 expression after treatment with FYCO1-LIR, **AM6**, and **AM10** (72 h and 96 h). Tubulin expression was evaluated as a protein control. Relative optical density was quantified by ImageJ software (version 1.54g). WB was performed independently three times, and a representative blot is presented. Data represent the mean values ± SEM and were analyzed by one-way analysis of variance ANOVA followed by Dunnett’s post hoc test (* *p* < 0.05 vs. C; ** *p* < 0.01 vs. C; *** *p* < 0.001 vs. C; **** *p* < 0.0001 vs. C).

In order to evaluate in more depth the ability of the compounds to act as autophagy inhibitors in PC-3 cells, the expression of the LC3 and SQSTM1 (sequestosome1, p62) proteins was analyzed. Treatment with FYCO1-LIR, **AM6**, and **AM10** for 48 h at a dose of 5 µM significantly reduced the level of LC3-I and LC3-II, without modifying the LC3-II/LC3-I ratio. It is plausible that all compounds bind to the LC3 precursor and prevent the efficient processing of LC3 by the cysteine protease Atg4, reducing the formation of both LC3-I and LC3-II without changing the ratio of LC3-II/LC3-I. This action inhibits the formation of autophagosomes and then the basal autophagy process. The analysis of LC3 expression after 72 h of treatment shows that FYCO1-LIR and **AM6** lose their efficacy, while **AM10** retains the ability to inhibit both LC3-I and LC3-II (Figure 6A). In addition, we analyzed the expression of p62, another protein involved in the autophagic process. This protein is recruited into autophagosomes linked to the material to be addressed for degradation in lysosomes. The p62 protein represents a marker of the autophagic flux; in fact, when autophagosomes fuse with lysosomes, the materials in autophagolysosomes were degraded including p62. The results obtained show that after treatment with the compounds for 72 h and 96 h, the expression of p62 increases significantly, demonstrating an impairment of autophagic flux. This result highlights that the compounds, mainly **AM10**, could determine an accumulation of materials that are not correctly degraded at the lysosomal level (Figure 6B). The analysis of the molecular mechanism suggests that the compounds interfere with the endogenous autophagy that preserves the tumor cells’ survival; furthermore, it is presumable that the inhibition of a pro-survival basal autophagy determines a stressful condition which leads to a reduction in cell viability as demonstrated by the MTT assays.

**Figure 7:**
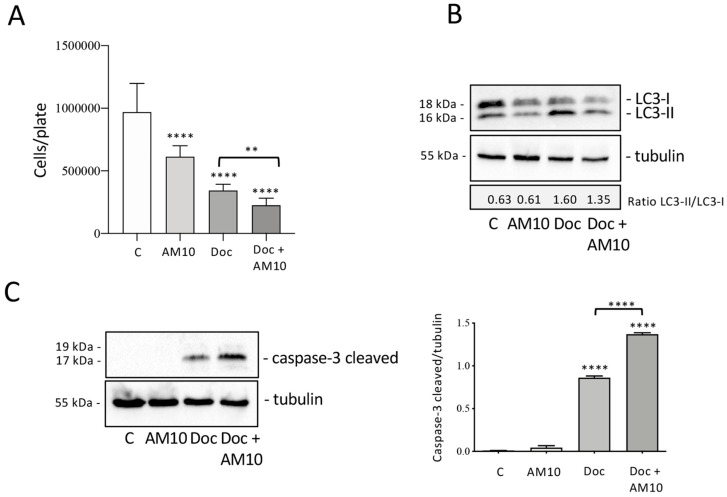
Effect of Docetaxel (**Doc**) in combination with AM10 on PC-3 cell proliferation and death. (**A**) PC-3 cells were simultaneously treated with **Doc** (25 nM) and **AM10** (5 mM) for 48 h. Cell growth was evaluated by cell count. Data represent the mean values ± SEM of four biological samples (n = 4) and were analyzed by one-way analysis of variance ANOVA followed by Tukey’s post hoc test (**** *p* < 0.0001 vs. C; ** *p* < 0.01). (**B**) Analysis of LC3 expression after treatment with **Doc** (25 nM) and **AM10** (5 mM) for 48 h. Tubulin expression was evaluated as protein control. (**C**) Analysis of caspase-3 cleaved after treatment with **Doc** (25 nM) and **AM10** (5 mM) for 48 h. The tubulin expression was evaluated as a protein control. WB was performed independently three times, and a representative blot is presented. Relative optical density was quantified by ImageJ software (version 1.54g). Data represent the mean values ± SEM and were analyzed by one-way analysis of variance ANOVA followed by Tukey’s post hoc test (**** *p* < 0.0001 vs. C).

An important problem of tumor is represented by therapy resistance [38]. In fact, in prostate cancer, numerous studies have investigated the implications of autophagy in resistance to hormonal therapies or chemotherapeutic agents. Abiraterone and Enzalutamide, currently employed in CRPC therapy, activate an autophagic response that reduces their effectiveness [39]. Docetaxel (**Doc**), a chemotherapeutic drug which inhibits microtubule depolymerization, represents the first-line treatment for metastatic CRPC. The treatment is effective in the early stages, but over time its efficacy is drastically reduced [40,41]. For this reason, a lot of research was focused on the molecular mechanisms involved in chemotherapy resistance including autophagy [21,22,42]. The action of **AM10** in combination with **Doc** was then analyzed in PC-3 cells to evaluate its ability to modulate the cytotoxic action of **Doc**. An MTT assay was performed to determine the dose of **Doc** capable of significantly reducing cell viability. PC-3 cells were treated with **Doc** at concentrations of 1 nM, 10 nM, 20 nM, 50 nM, and 100 nM, for an incubation time of 48 h. The results showed a significant reduction in cell viability at concentrations of 10 nM, 25 nM, 50 nM, and 100 nM, with a dose-dependent effect. The effect of simultaneous treatment with **Doc** (25 nM) and **AM10** (5 µM) on cell growth was subsequently evaluated by cell count. The results obtained showed that treatment with **AM10** increases the antitumoral activity of **Doc** in a significant manner (Figure 7A). The impact of **AM10** on **Doc**-induced autophagy activation was then explored by analysis of LC3 expression. A Western blot of LC3 shows that **AM10** treatment does not determine a change in the LC3-II/LC3-I ratio but reduces the expression of both LC3-I and LC3-II. On the contrary, **Doc** treatment determines a significant increase in LC3-II expression, indicative of autophagy activation in response to the stress induced by the compound. Combination treatment conducted simultaneously for 48 h with **Doc** and **AM10** determines a reduction in LC3-II expression compared to **Doc** alone, suggesting that **AM10** can inhibit **Doc**-induced autophagy (Figure 7B). Then, **AM10** in combination with **Doc** can counteract the autophagic activation induced by **Doc**, potentially influencing the sensitivity of PC-3 cells to chemotherapy.

It is known that autophagy and apoptosis are interconnected phenomena, and several studies have examined how autophagy may influence the ability of **Doc** to trigger apoptotic cell death on PC cells. The observations obtained are still discordant, and to date it is impossible to draw conclusions on the role of autophagy in the regulation of **Doc** cytotoxicity and in the development of resistance in CRPC cells [43,44,45,46]. For this reason, we evaluated the activation of apoptosis by analysis of caspase-3 cleavage after simultaneous treatment with **Doc** and **AM10**. Figure 7C highlights that **AM10** does not induce caspase-3 activation, unlike **Doc** which is known to trigger apoptotic cell death by activating the executor caspase-3. The combined treatment enhances the expression of cleaved caspase-3, demonstrating that the inhibition of **Doc**-induced autophagy by **AM10** enhances the apoptotic cell response. Therefore, **AM10** acts as sensitizing chemotherapy rather than directly promoting apoptosis (Figure 7C). Consequently, we can affirm that **AM10**, by inhibiting the pro-survival autophagy induced by **Doc**, could represent a therapeutic opportunity to enhance the efficacy of **Doc** and decrease the dose of taxanes that are responsible for numerous side effects and reduce the resistance to this chemotherapy in CRPC.

Many studies have analyzed the nature of autophagy activated by **Doc** in PC and the impact of autophagy inhibitors on cell proliferation. The results obtained using 3-Methyladenine (3-MA) in association with **Doc** were contradictory. Hu and collaborators reported how 3-MA enhanced the cytotoxic action of **Doc** [46], while other studies showed that 3-MA decreased the chemotherapy efficacy [43,47,48]. Finally, our study showed that 3-MA does not modify the cytotoxicity of **Doc** in PC-3 cells [45]. A more convincing result was obtained using the autophagy inhibitor CQ which enhanced the action of **Doc** by reducing resistance to chemotherapy [49].

**Figure 8:**
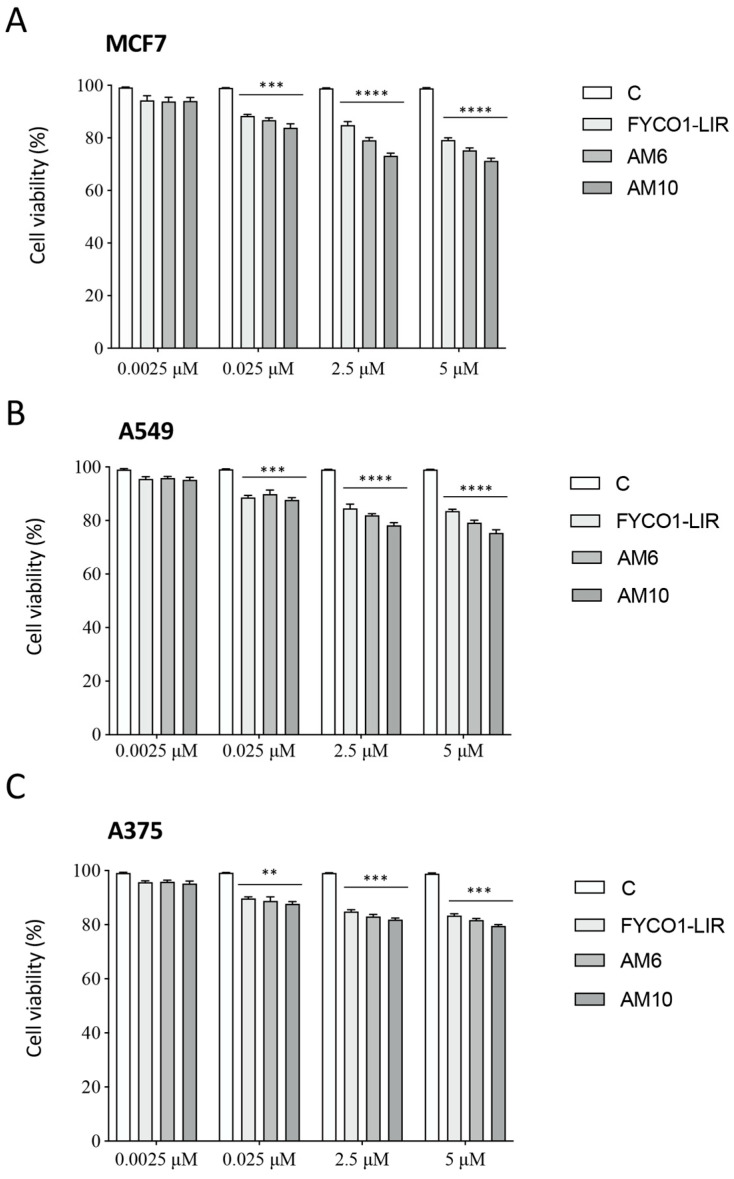
Effect of FYCO1-LIR analogs on different cancer cell lines. MCF-7 (**A**), A549 (**B**), and A375 (**C**) cells were treated with FYCO1-LIR, **AM6**, and **AM10** for 72 h. Cell viability was evaluated by MTS assay. Data the represent the mean values ± SEM of four biological samples (n = 4) and were analyzed by one-way analysis of variance ANOVA followed by Tukey’s post hoc test (** *p* < 0.01 vs. C; *** *p* < 0.001 vs. C; **** *p* < 0.0001 vs. C).

***Biological assays on different cancer cell lines***. The cell viability of different concentrations of FYCO1-LIR, **AM6**, and **AM10** (from 0.0025 to 5 μM) peptides was evaluated with an MTS assay on the MCF-7, A549, and A375 cancer cell lines. Interestingly, as shown in Figure 8, a concentration-dependent reduction in cell viability (expressed as percentage % of viable cells) was observed in all tested cell lines as compared to the untreated control cells. In more depth, the results showed that MCF-7 cells are more responsive than the other tested cell lines. Overall, these findings support the effectiveness of these compounds in tumor cell lines with various origins and characteristics (Figure 8). Additionally, considering these results suggests that the AM10 peptide could have interesting application in cancer therapy; nevertheless, further studies should be conducted to better understand the translational aspects of the tested therapy.

## 3. Materials and Methods

### 3.1. Computational Design of FYCO1 Analogs

The starting computational model of LC3B was generated using the 3D coordinates from the LC3B/FYCO1-LIR complex (PDB accession code 5D94 [26]). The complex model was optimized using the Protein Preparation Wizard in Maestro (release 2021–2, Schrödinger, LLC, New York, NY, USA), which included the following: residue protonation state assignment at pH 7.4, residue verification, clash resolution, and application of the OPLS4 force field. Then, the protein-ligand complex was solvated in a cubic box of TIP3P water molecules and subjected to energy minimization, followed by 250 ns MD simulations using the Desmond algorithm of Maestro (release 2021–2, Schrödinger, LLC, New York, NY, USA) [50]. The peptide stability in complex with LC3B was assessed using the “Simulation Interactions Diagram” tool. The Cα atoms RMSD and RMSF graphs of all the simulated systems are available in the Supplementary Materials, Figure S1 and Figure S2, respectively. The peptide binding free energy value was calculated using the Prime MM-GBSA algorithm in Maestro (release 2021–2, Schrödinger, LLC, New York, NY, USA) [51], employing the single-trajectory approach. The resulting binding free-energy value was designated as ∆G* [24,31] and calculated for all peptides (Table 1). The peptides of the AM series investigated in this paper (Table 1) were manually built starting from the LC3B/FYCO1-LIR complex and using the “mutate residue” and drawing tools available in Maestro (release 2021–2, Schrödinger, LLC, New York, NY, USA). The ∆G* values of the FYCO1-LIR analogs were calculated using the computational protocol adopted for the reference FYCO1-LIR peptide. The LC3B/Comb1 complex was generated through a three-step protocol:

1. Structural Alignment: The LC3B/FYCO1-LIR (PDB: 5D94 [26]) and LC3A/FYCO1 (PDB: 5CX3 [52]) X-ray structures were superimposed to establish a common spatial framework.
2. Peptide Transfer and Adaptation: The DDAVFDIITDEELCQIQESG peptide from the LC3A/FYCO1 complex was transferred onto the LC3B surface. This transfer was feasible because the FYCO1 segment (DAVFDIITDEEL) in the LC3B complex aligned perfectly with its counterpart in the LC3A structure. The sequence was then manually adjusted to match Comb1.
3. Refinement: The resulting LC3B/Comb1 model was optimized using energy minimization and MD simulations, following the protocol previously described.

### 3.2. Synthesis of Peptides

All material and reagents were purchased by Sigma Aldrich, if not otherwise stated. Peptides were assembled on a 2-CTC resin by stepwise solid phase Fmoc-chemistry in a 0.15 mmol scale. Concentrations of 0.5 M Oxyma and 0.5 M DIC were used as activators, while a 20% piperidine solution in DMF was used for Fmoc-removal. Upon iterative chain assembly, peptides were cleaved off the resin by treatment with a TFA-based mixture (92.5% TFA, 2.5% TIS, 2.5% thioanisole, 2.5% water), and precipitated in cold ether. Crude peptides were recovered by centrifugation, and HPLC-purified (C-18 column, Phenomenex). For cyclization purposes, peptides were oxidized by dissolution at 100 mM in a phosphate buffer (pH = 7.8), where 1.1 eq. of 10 mM H_2_O_2_ were added. The reaction was HPLC-monitored until completion and the resulting product HPLC-purified. For linker-based cyclization, to peptide solutions in 50:50 NaHCO_3_ aq./acetonitrile (pH = 8.0), 1.5 eq. of bis-alkylating reagent was added. The reaction was HPLC-monitored until completion and the resulting product HPLC-purified. The mass spectra and HPLC graphs of the FYCO1-LIR, **AM2**, **AM6**, **AM7**, and **AM10** peptides are available in the Supplementary Materials, Figure S3 and Figure S4, respectively.

### 3.3. Biophysical Assays

The interaction between peptides (FYCO1-LIR, **AM2**, **AM6**, **AM7**, **AM10**) and the LC3B protein was evaluated using the Monolith NT.115^Pico^ instrument (NanoTemper Technologies GmbH, München, Germany). This technique enables the determination of the dissociation constant (K_d_) across a concentration range spanning from 1 picomolar (pM) to the millimolar (mM) level. The experimental conditions mirrored those in our previously published work [31]. In summary, His-tagged human recombinant LC3B (Catalog No. 14555-H07E, Sino Biological, Beijing, China) was labelled with the His-Tag Labelling Kit RED-tris-NTA 2nd Generation (Product No. MO-L018, NanoTemper Technologies GmbH, München, Germany) for 30 min at room temperature. A constant concentration of red-labelled LC3B (10 nM) was incubated with sixteen 1:1 serial dilutions of peptides (concentration details are consultable in the Supplementary Materials, Table S1). PBS-T (phosphate-buffered saline + 0.05% Tween™ 20), from NanoTemper Technologies, with 2.5% dimethyl sulfoxide (DMSO) (Product No. D8418; Sigma-Aldrich, Saint Louis, MO, USA) was used as buffer. After 15 min of incubation at room temperature, samples were loaded into standard capillaries (Product No. MO-K022; NanoTemper Technologies) and analyzed at 25 °C with 20% excitation power and medium MST power (40%) using the “Binding Affinity” mode available in the MO.Control v1.6 software (NanoTemper Technologies GmbH, München, Germany). Prior to K_d_ determination, peptide auto-fluorescence was assessed. Data analysis was performed using MO.Affinity Analysis v2.3 software (NanoTemper Technologies GmbH, München, Germany) applying the K_d_ model for fitting the binding curve, while the figures were generated using GraphPad Prism v8.0.2 software (GraphPad, Boston, MA, USA). In the case of **AM7**, the three highest concentrations (125, 62.5, and 31.25 µM) were excluded from analysis due to aggregation phenomena, as evidenced in the Supplementary Materials, Figure S5D. The “Capillary Scan” graphs, showing the fluorescence homogeneity in the capillaries of all biophysically tested peptides, are provided in the Supplementary Materials, Figure S5.

### 3.4. Cell Lines

The study was conducted on several cancer cells. Human CRPC cell lines (PC-3 and DU145) were purchased from the American Type Culture Collection (ATCC, Manassas, VA, USA) and cultured at 37 °C and 5% CO_2_ in RPMI 1640 (EuroClone, Milano, Italy) supplemented, respectively, with 7.5% (PC3) and 5% (DU145) FBS (Gibco, ThermoFisher Scientific, Waltham, MA, USA), 1% L-glutamine, and antibiotics (100 IU/mL penicillin G). Luminal A MCF-7 breast cancer cells (ATCC, Manassas, VA, USA) were cultured in Dulbecco’s Modified Eagle Medium/Nutrient Mixture F-12 (DMEM-F12, Gibco Laboratories, Waltham, MA, USA) supplemented with 10% fetal bovine serum (FBS, Gibco Laboratories, Waltham, MA, USA), 1% penicillin/streptomycin (Gibco Laboratories), and 1% L-glutamine (Gibco Laboratories). A549 human lung cancer cell line (ATCC, Manassas, VA, USA) were cultured in Dulbecco’s modified eagle medium (DMEM, Lonza, Switzerland) supplemented with 10% FBS (Gibco Laboratories, Waltham, MA, USA), 1% of 100 u/mL penicillin/streptomycin (Gibco Laboratories), and 1% L-glutamine (Gibco Laboratories). The A375 melanoma cell line (ATCC, Manassa, VA, USA), derived from skin lesions was cultured in RPMI 1640 media (Gibco Laboratories, Waltham, MA, USA) supplemented with 1% of penicillin/streptomycin (Gibco Laboratories, Waltham, MA, USA), 1% L-glutamine (Gibco Laboratories, Waltham, MA, USA), and 10% FBS (Gibco Laboratories).

### 3.5. Cell Viability Studies

For viability studies, MTS (Cell Titer 96 Aqueous One Solution Cell Proliferation Assay) (Promega, Nacka, Sweden) and 3-(4,5-dimethylthiazole-2-yl)-2,5-diphenyltetrazolium bromide (MTT) (Sigma-Aldrich, St. Louis, MO, USA) assays were conducted. PC-3 and DU145 cells were plated at the density of 3 × 10^4^ cells/well in 24-well plates. After 48 h, cells were treated with FYCO1-LIR, **AM6**, and **AM10** at 0.0025 μM, 0.025 μM, 0.25 μM, 2.5 μM, and 5 μM doses for 72 h. At the end of the treatments the medium was replaced with MTT solution (0.5 mg/mL) in RPMI without phenol red and FBS. Following 30–45 min of incubation at 37 °C, the precipitate was dissolved with isopropanol. The absorbance (λ = 550 nm) was measured by an EnSpire Multimode Plate reader (Perkin Elmer, Milano, Italy). The absorbance value of untreated cells was set at 100% (control), and the viability of treated cells was expressed as a percentage of the control. Three independent experiments were performed for each condition. MCF-7, A549, and A375 cells were seeded at a density of 1 × 10^4^ cells/well in 96-well plates and maintained under standard growth conditions. After 24 h, the cells were treated with FYCO1-LIR, **AM6** and **AM10** at 0.0025 μM, 0.025 μM, 2.5 μM, and 5 μM doses. After 72 h, cell viability was assessed using an MTS assay according to the manufacturer’s protocol using a 96-well-plate spectrophotometer (Varioskan Flash Multimode Reader; ThermoFisher Scientific, Waltham, MA, USA) set at λ = 490 nm. The absorbance value of untreated cells was set at 100% (control), and the viability of treated cells was expressed as a percentage of the control. Three independent experiments were performed for each condition.

### 3.6. Western Blot (WB) Assay

PC-3 cells were plated at 2 × 10^5^ cells/dish in 6 cm dishes and treated with different compounds. At the end of the treatments the cells were lysed in RIPA buffer. Protein extracts (15–35 μg) were resuspended in presence of reducing Sample buffer (Bio-Rad Laboratories, Segrate, Milano, Italy), heated at 95 °C for 5 min and separated by SDS-PAGE WB. Proteins were transferred onto nitrocellulose or PVDF membranes. After blocking with nonfat dried milk, membranes were incubated with anti-ATG5 (1:1000) (#12994) Cell Signaling Technology Inc. (Boston, MA, USA), anti-LC3 (1:1000) (L8918) (Sigma-Aldrich), anti-SQSTM1/p62 (1:2000) (PA5-20839) (Thermo Fisher Scientific, Waltham, MA, USA), anti-cleaved-caspase-3 (1:500) (#9664) Cell Signaling Technology Inc. primary antibodies overnight at 4 °C. Horseradish peroxidase (HRP) conjugated secondary anti-rabbit or anti-mouse antibodies were used for 1 h at room temperature and the membranes were processed using chemiluminescence kit Cyanagen Ultra (Cyanagen, Bologna, Italy). In each WB experiment alpha-tubulin expression (T6199) (Sigma-Aldrich, St. Louis, MO, USA) was evaluated as a protein control. Relative optical density of the bands was assessed by ImageJ software (version 1.54g). Uncropped WB images are available in the Supporting Information.

### 3.7. Cell Proliferation Studies

PC-3 cells were plated at 2 × 10^5^ cells/dish in 6 cm dishes and treated simultaneously with **AM10** (5 mM) in combination with **Doc** (25 nM) for 48 h. The cells were then collected and counted using a hemocytometer.

### 3.8. Statistical Analysis

Statistical analysis of the results was performed by one way performed of variance (ANOVA) followed by Dunnett’s test or Tukey’s multiple comparison post-test. Prism software was used for the analyses (Prism 8 for Mac OS version 8.2.1, GraphPad Software, San Diego, CA, USA).

## 4. Conclusions

This work began by studying the sequence of FYCO1-LIR, a peptide known to bind LC3B, and applied a computational approach combining MD simulations and MM-GBSA calculations to predict the binding affinity of novel peptides generated through sequence mutations. Our methodology significantly enhanced both the stability and affinity of the redesigned peptides (Table 1, Figure 1 and Figure 2). The electrostatic interactions between the new peptides and LC3B’s positively charged surface—defined by the residues K49, K51, R69, and R70—correlated with low predicted ΔG* values, explaining their improved binding efficiency. Structural rigidity conferred by disulfide bridges in the peptide sequences reduced fluctuations at LC3B’s LIR binding site while enhancing the occupancy of the HP1 hydrophobic basin (F7, I23, P32, I34, L53, and F108). Notably, the top analog, **AM10** (a decamer), demonstrated a binding mode comparable to Comb1 (eicosapeptide, K_d_ = 0.12 µM), despite its shorter length [30]. Experimental validation via MST confirmed **AM10**′s superior affinity (K_d_ = 0.04 µM), representing an approximately 80-fold improvement over the parent FYCO1-LIR peptide (K_d_ = 3.1 µM). Biological assays in PC-3 cells revealed that **AM6** and **AM10** diminished cell viability and inhibited autophagosome formation and autophagic flux, as evidenced by the expression of LC3-I, LC3-II, and p62. Furthermore, co-treatment with **Doc** and **AM10** determined an enhancement of apoptosis, demonstrating that **AM10** is able to counteract **Doc**-induced autophagy which causes a reduction in chemotherapy-induced apoptotic cell death. Overall, the results obtained may suggest the use of **AM10** to reduce resistance to pharmacological therapies that activate a cellular protective pro-survival autophagic response.

The efficacy of **AM10** against prostate cancer and other different tumoral cells suggests its use as a novel autophagy modulator for cancer treatment, particularly if intracellular delivery via nanocarriers or liposomes is optimized. Moreover, since **AM10** can bind to the LC3B area close to the arginine-rich motif (residues 68 to 70), the one regulating the mRNA degradation during autophagy [53], our results open the way to design new peptide nucleic acids (PNAs) for RNA-based therapeutics, which could represent a new class of autophagy modulators.

## Figures and Tables

**Table 1:** Primary structure and estimated binding free energy values (ΔG*) of the reference peptide FYCO1-LIR and its analogs. Disulfide bonds are represented as square brackets (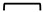). The curly bracket (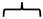) of **AM7** joins two cysteines bis-alkylated by a para-dibromomethylbenzene (see the Materials and Methods section for details).

Peptide	Sequence	ΔG* (kcal/mol)	SD (kcal/mol)	Avg. Cα RMSF (Å)
**FYCO1-LIR**	DAVFDIITDEEL	−110.6	7.0	1.49
**Sequence mutation**				
**AM1**	DAVFDI**M**TDEEL	−113.0	7.3	1.52
**AM2**	DAV**F**_**I**_DIITDEEL	−119.2	10.1	1.29
**AM3**	DAV**F**_**Br**_DIITDEEL	−111.6	7.7	1.44
**AM4**	DAV**F**_**I**_DI**M**TDEEL	−123.8	5.8	1.13
**Backbone rigidification**				
**AM5**	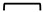 DAVFDIIT**C**EE**C**	−109.5	6.2	1.26
**AM6**	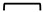 DAVFDI**M**T**C**EE**C**	−126.6	8.4	1.38
**AM7**	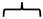 DAVFDIIT**C**EE**C**	−118.5	8.3	1.00
**AM8**	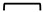 DAV**F**_**I**_DIIT**C**EE**C**	−109.2	5.3	1.03
**AM9**	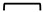 DAV**F**_**Br**_DIIT**C**EE**C**	−110.4	7.3	1.17
**AM10**	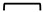 DAV**F**_**I**_DIMT**C**EE**C**	−137.1	6.1	0.60

## Data Availability

Data are contained within the article and Supplementary Materials.

## Supplementary Materials

The following supporting information can be downloaded at https://www.mdpi.com/article/10.3390/ijms26115365/s1.
