# Supplementary material for: FYCO1 Peptide Analogs: Design and Characterization of Autophagy Inhibitors as Co-Adjuvants in Taxane Chemotherapy of Prostate Cancer
Source: Int J Mol Sci. 2025 Jun 3;26(11):5365. doi: 10.3390/ijms26115365 (PMC12154284; doi:10.3390/ijms26115365)
Supplement: Supplementary file 1 [file ijms-26-05365-s001.zip › ijms-3657995-supplementary.pdf]

Supplementary Materials

FYCO1 Peptide Analogs: Design and Characterization of Autophagy Inhibitors as Co-Adjuvants in Taxane Chemotherapy of Prostate Cancer

Enrico Mario Alessandro Fassi [1,*], Roberta Manuela Moretti [2], Marina Montagnani Marelli [2], Mariangela Garofalo [3], Alessandro Gori [4], Cristiano Pesce [3], Marco Albani [1], Erica Ginevra Milano [1], Jacopo Sgrignani [5], Andrea Cavalli [5] and Giovanni Grazioso [1]

[1] Department of Pharmaceutical Sciences, Università degli Studi di Milano, Via L. Mangiagalli 25, 20133 Milano, Italy

[2] Department of Pharmacological and Biomolecular Sciences, Università degli Studi di Milano, Via G. Balzaretti 9, 20133 Milano, Italy

[3] Department of Pharmaceutical and Pharmacological Sciences, Università di Padova, Via F. Marzolo 5, 35131 Padova, Italy

[4] National Research Council of Italy, Istituto di Scienze e Tecnologie Chimiche (SCITEC-CNR), Via M. Bianco 9, 20131 Milano, Italy

[5] Institute for Research in Biomedicine (IRB), Via Chiesa 5, 6500 Bellinzona, Switzerland

*Correspondence: enrico.fassi@unimi.it

Table S1. Dataset overview of Microscale Thermophoresis (MST) experiments conducted with a fixed concentration of human recombinant His-tagged LC3B protein (10 nM) and varying concentrations of FYCO1-LIR peptide (positive control) along with its peptide analogs. Two independent experiments were performed to determine the K_d value.

Peptide	MST Power	Exc. Power	Temp.	[Ligand] Range (µM)	Time	RA	SNR	K_d (nM)
FYCO1-LIR	40%	20%	25 °C	500 – 0.0305	5 s	4.3	10.4	3592 ± 1159
AM2	40%	20%	25 °C	250 – 0.00763	5 s	7.8	14.4	2163 ± 486
AM6	40%	20%	25 °C	125 – 0.00381	2.5 s	3.2	8.7	615 ± 228
AM7	40%	20%	25 °C	125 – 0.00381	5 s	3.7	9.1	869 ± 384
AM10	40%	20%	25 °C	39.1 – 0.00119	1.5 s	2.8	12.8	38 ± 10

RA = Response Amplitude; SNR = Signal-to-Noise Ratio.

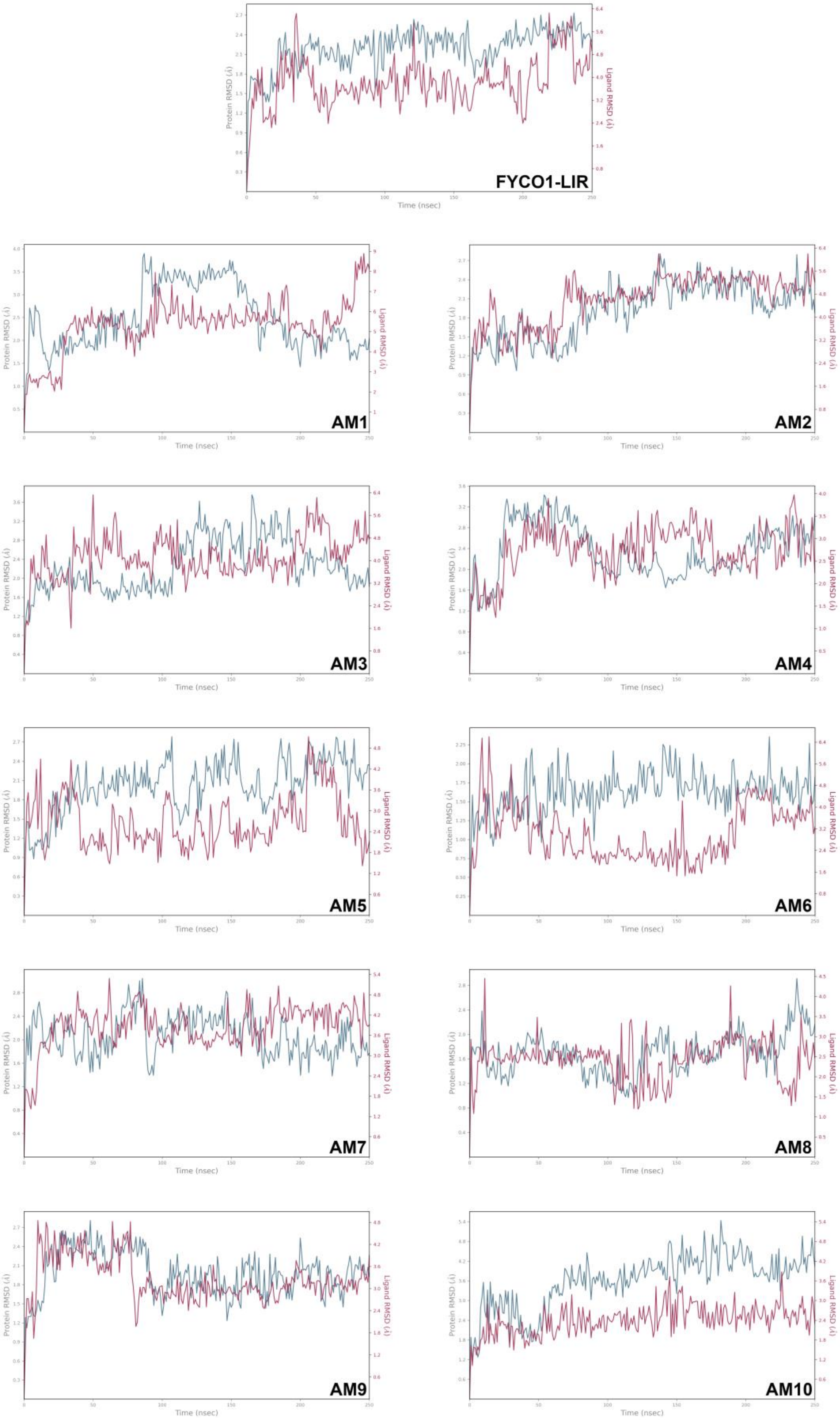

Figure S1. Cα atoms RMSD plots of all the simulated systems over 250 ns of MD simulation time.

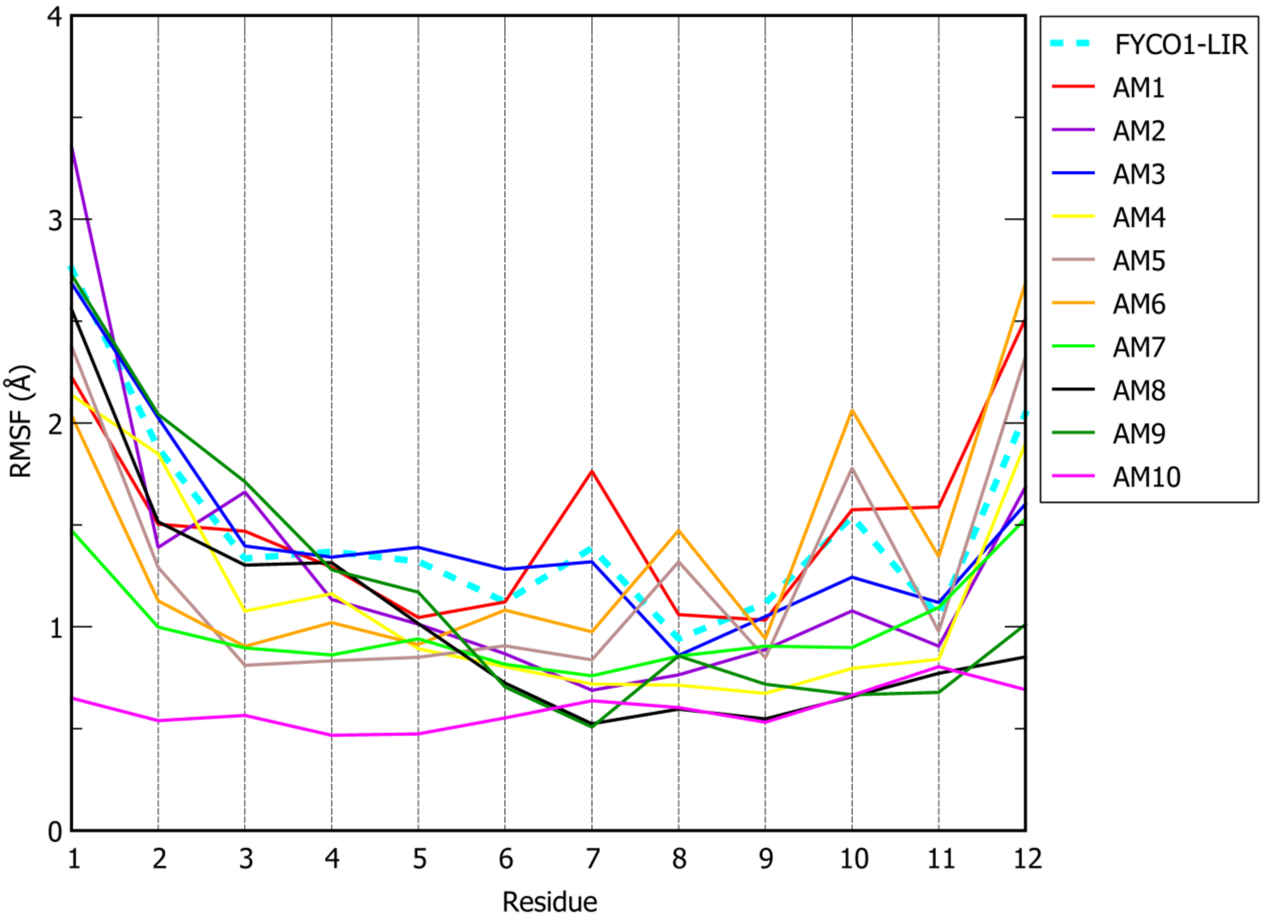

Figure S2. Cα atoms RMSF plots of all the simulated systems over 250 ns of MD simulation time.

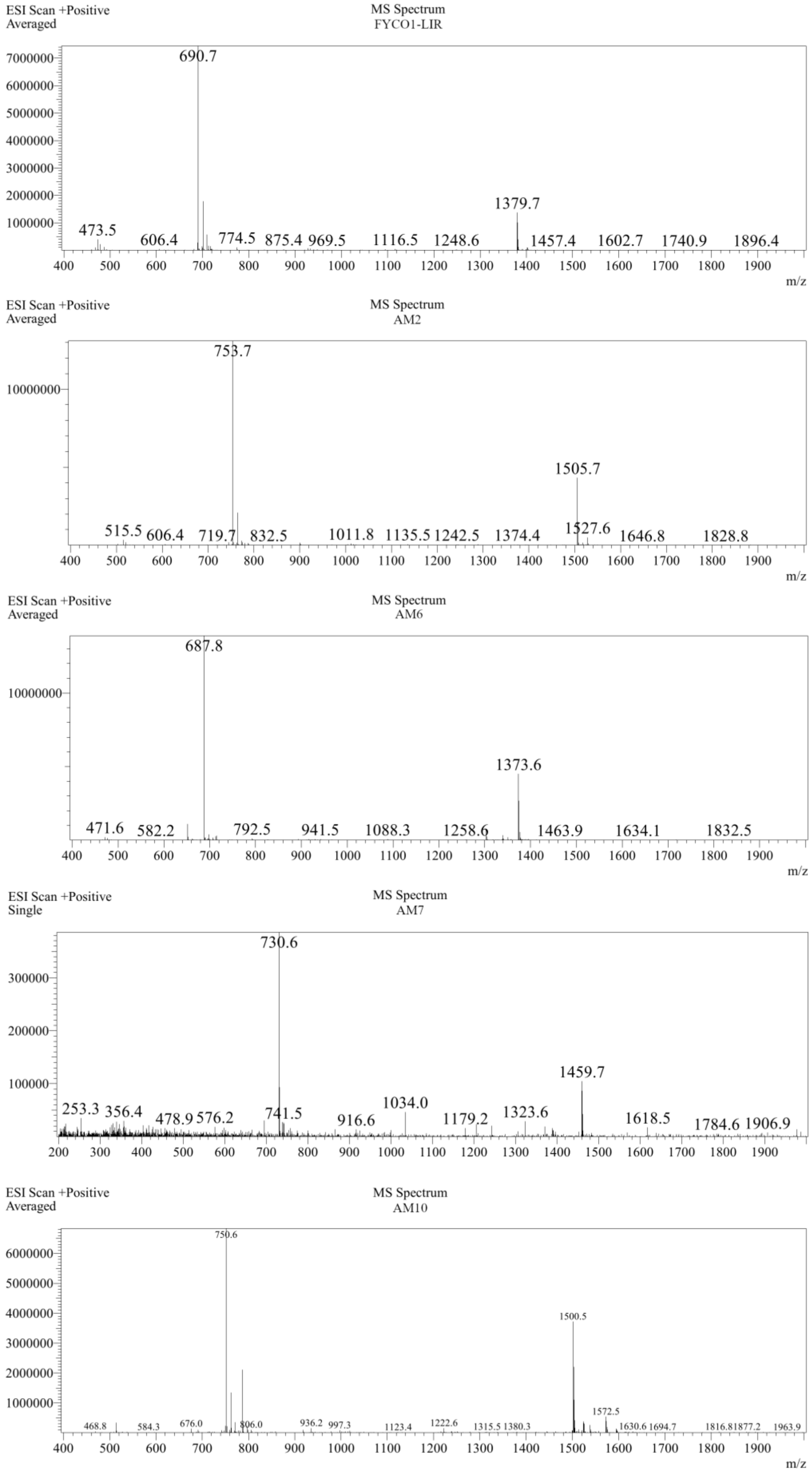

Figure S3. Mass spectra of the synthesized peptides chosen for biophysical experiments.

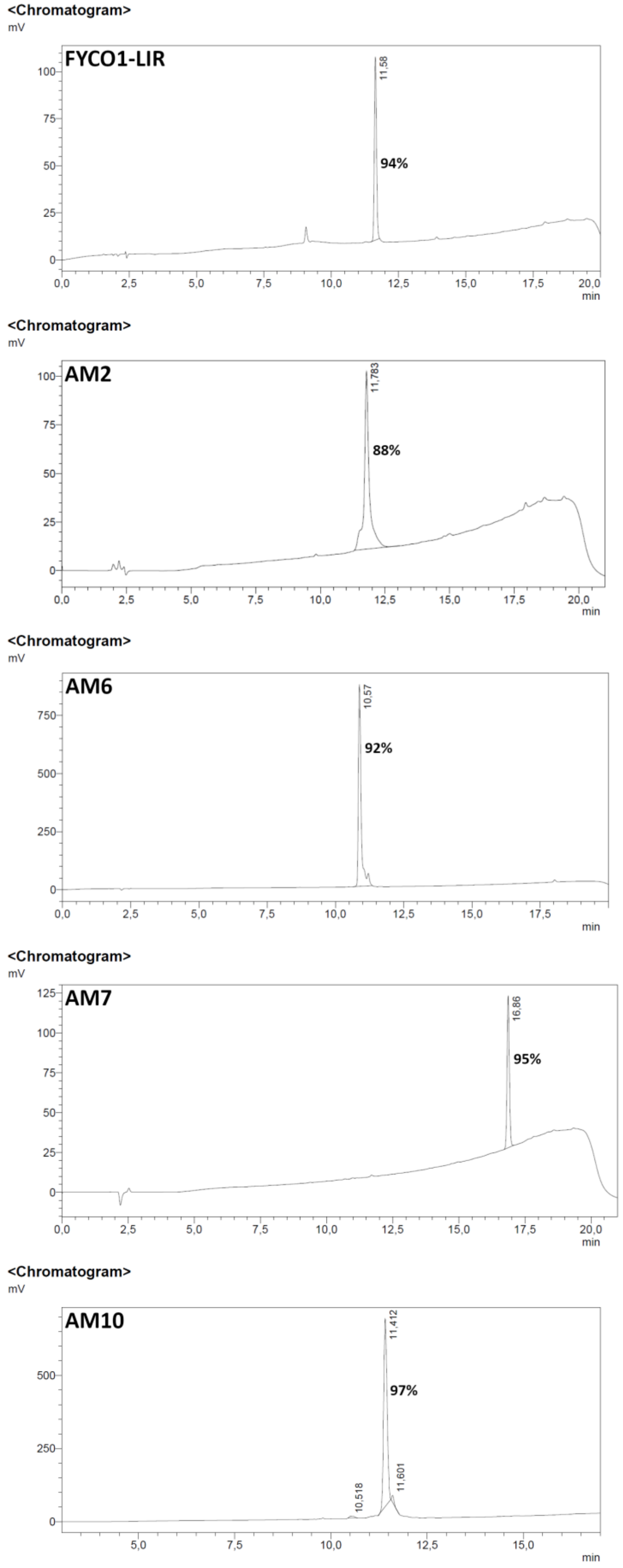

Figure S4. HPLC graphs with purity value of the synthesized peptides chosen for biophysical experiments.

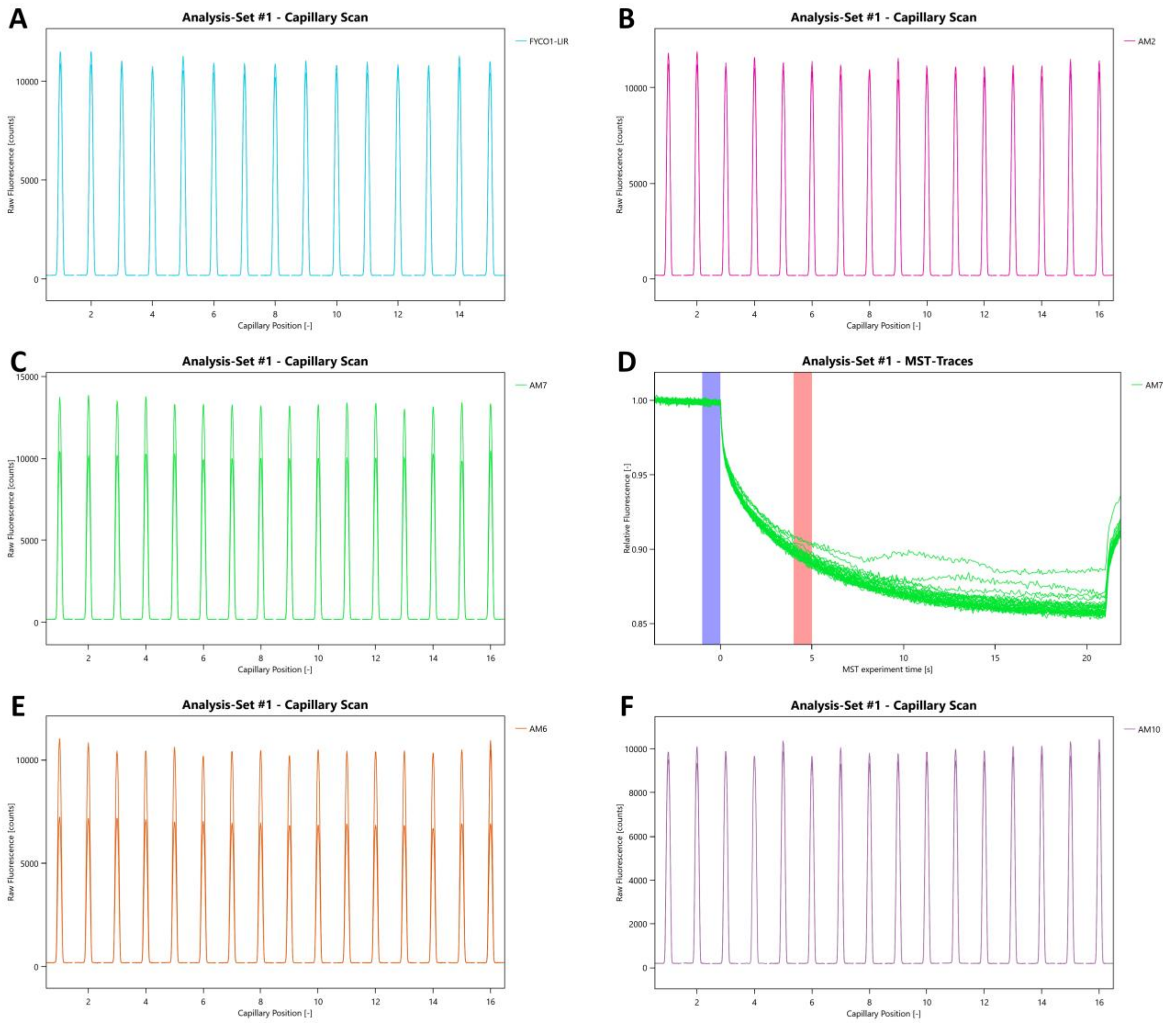

Figure S5. "Capillary Scan" graphs displaying the overall fluorescence intensities in the different samples in the case of (**A**) FYCO1-LIR, (**B**) **AM2**, (**C**) **AM7**, (**E**) **AM6** and (**F**) **AM10** peptides. (**D**) "MST Traces" graph of **AM7**, in which aggregation phenomena can be observed at the three highest concentration points (125, 62.5, and 31.25 µM).
